# News and Coming Events

**Published:** 1908-08-15

**Authors:** 


### News and Coming Events.

Mr. Aslett Baldwin, F.R.C.S., has been appointed assistant surgeon to St. Mark's Hospital for Diseases of the Rectum.

Dr. C. M. Hinds Howell, M.B., M.R.C.P., has been appointed physician to the Great Northern Central Hospital.

Mr. J. Henry Chaldecott, L.R.C.P., F.F.P.S., has been appointed honorary anæsthetist to St. Mary's Hospital, London, W.

Mr. Cecil Graham, F.R.C.S., has been appointed assistant surgeon in charge of the new Throat, Nose, and Ear Department of St. Mary's Hospital.

Dr. J. Booth Clarkson, L.R.C.P., serving in the Colonial Civil Service, has been appointed health officer and medical officer at Ayr, Queensland, in place of Dr. Arthur Cole, who has resigned.

The winter session of the Charing Cross Hospital Medical School will be opened on October 1, when Sir Patrick Manson will deliver the Seventh Biennial Huxley Lecture on "Recent Advances in Science and their Bearing on Medicine and Surgery."

At the Amalgamated Hampstead General and North-West London Hospitals, Mr. Harold Barwell, M.B., F.R.C.S., has been appointed surgeon to the Throat, Nose, and Ear Department; Dr. S. Ernest Dore, M.B., M.R.C.P., physician to the skin department; and Dr. Frank E. Taylor, M.D., M.R.C.P., F.R.C.S., gynæcologist.

Mr. George Allardice MacDonald, M.B., C.M.Edin., has received the King's permission to accept and to wear the insignia of the third class of the Order of El Aliyeh, which has been conferred upon him by his Highness the Sultan of Zanzibar in recognition of valuable services rendered by him.

Surgeon-General W. Launcelotte Gubbins, C.B., M.V.O., who was Chief Medical Officer of the Home District from August 1903 until appointed Principal Medical Officer in India, has been transferred to the War Office headquarters as Deputy Director-General of the Army Medical Service.

The Metropolitan Asylums Board is handing over the Belmont Asylum, near Banstead, to be used as a workhouse for the accommodation of inmates from overcrowded London workhouses, and the Fulham Guardians have undertaken the administration of the new workhouse on condition that they are to make no profit out of the undertaking and suffer no pecuniary loss.

At the last meeting of the Council of the Royal College of Surgeons of England, Mr. Clinton T. Dent gave notice that at the next meeting of the Council he would move :—(1) That steps be forthwith taken to admit women to the examinations of the Conjoint Examining Board of England and to the examination for the Diploma in Public Health. (2) That women be admitted to the examinations for the Fellowship of the College and to the examinations for the license in dental surgery.

The death is announced of Brigade-Surgeon Melville George Jones at the age of seventy-three. He qualified M.R.C.S. in 1856, and joined the Army Medical Department. In the Jowaki campaign in 1877 he served with the 51st Light Infantry, receiving a medal with clasp, and also in the Afghan War of 1878-80. For his services during the last-mentioned campaign he was awarded a medal.

A decree has recently been enacted by the French Government whereby the composition of various beverages and other liquids is to be controlled and regulated. Thus the amount and nature of the antiseptics that may be contained in beer are defined, and the artificial aëration of cider is restricted. Vinegar made from wine or malt and vinegar made from wood are to be distinguished under penalty, and liqueurs and syrups must conform to the Government standard if they are to be sold as such. It is also forbidden to sell under the name of cognac *eau-de-vie* from any other region than the Charentes; and no sparkling wine, however good, may be styled champagne which is made from grapes grown in any province other than Champagne.

On the morning of August 8 his Majesty the King, with Queen Alexandra, the Prince of Wales, Princess Victoria, and Prince Edward of Wales, with a large suite, paid a surprise visit to the Royal Naval Hospital at Haslar. The Royal party crossed the harbour in the yacht's pinnaces, and walked through the avenue from the landing stage to the hospital, where only the briefest notice of their visit had preceded them. At the institution their Majesties were received by Inspector-General T. D. Gimlette and the Staff, and were conducted through the building. The Royal party first visited the laboratory, where Fleet-Surgeon Bassett-Smith, who has charge of this important experimental department, explained the nature of the work carried on there. The building has only been constructed a few years. Considerable time was spent in the laboratory, and then the distinguished party visited the officers' sick quarters, and passed through two of the men's wards. At the main entrance the invalid's tramway awaited the Royal party and conveyed them to the boats.

### Child Rescue.

Under the title of "A Chart of Child-Rescue" Dr. Barnardo's Homes enter upon a new departure in their annual report for 1907, just being issued. This is a handsome illustrated quarto pamphlet of sixty-four pages. Its special feature is a large map of England and Wales, on which are marked in red the towns, cities, villages, and districts from which during the last twenty-four years nearly 40,000 children have been admitted to the homes. The map is excellently printed, and is not attached to the rest of the publication. It proves, and it ought to keep before the public, the really national claims of these celebrated institutions. The homes have been in existence forty-two years, and they have now 136 branches, by means of which they meet the needs of juvenile destitutes throughout the kingdom. Among their 8,000 inmates there are 1,100 babies and 1,050 of the deformed, crippled, helpless, or incurable class. No conditions are laid down for admission beyond destitution. The report forms a human document of the deepest interest, and it is well worth studying by all who have at heart the problem of the destitute child. A copy of the report can be obtained post free for six penny stamps on application to the hon. secretary at the headquarters of the homes, 18 to 26 Stepney Causeway, London, E.

It is announced that Lieut.-Colonel C. H. Melville, M.B., C.M., D.P.H., Secretary and Expert in Sanitation of the Army Medical Advisory Board, is to be appointed Professor of Hygiene at the Royal Army Medical College.

The Home Secretary has extended the reference to the Departmental Committee upon the operation of the law relating to inebriates and to their detention in reformatories and retreats, and has authorised the Committee to investigate the value of existing methods for the treatment of inebriety by the use of drugs.

While driving in his carriage on Thursday, August 6, Dr. W. W. Andrew, M.B., M.R.C.S., of Hendon, was stung in the face by gnats, and this was followed by erysipelas which ended fatally. He had formerly been attached to the Army Medical Service, and upon his retirement he was appointed vaccination officer, divisional police surgeon, and Union medical officer at Hendon. He qualified in 1860, and had been house surgeon at St. Bartholomew's Hospital.

In the House of Commons Mr. Gladstone recently stated in reply to a question that the number of deaths under anæsthetics, administered for the purpose of operations, which had occurred in England and Wales were, in 1901, 133; in 1902, 148; in 1903, 146; in 1904, 156; in 1905, 155; and in 1906, 183. He further stated in writing that he was in communication with the Lord President of the Council, and through him with the General Medical Council, with regard to the question whether a course of instruction on the administration of anæsthetics could be included in all cases in the course of study required for medical examinations.

The King has been pleased to make the following appointments: David W. Finlay, Esq., M.D., F.R.C.P., London, Professor of the Practice of Medicine in the University of Aberdeen, to be one of the Honorary Physicians to his Majesty in Scotland, in the room of Sir Thomas McCall Anderson, M.D., deceased. Sir William Macewen, F.R.S., M.D., Regius Professor of Surgery in the University of Glasgow, to be one of the Honorary Surgeons to his Majesty in Scotland, in the room of Sir Patrick Heron Watson, M.D., deceased. James Little, Esq., M.D., Regius Professor of Physic in the University of Dublin, to be one of the Honorary Physicians to his Majesty in Ireland, in the room of Sir John Thomas Banks, K.C.B., M.D., deceased. William Black Alexander, Esq., to be Surgeon Apothecary to his Majesty's Household at Holyrood Palace

At the forthcoming Dublin meeting of the British Association the Physiology Section will be presided over by Dr. J. S. Haldane, Reader in Physiology in the University of Oxford. As the subject of his presidential address he has selected "The Relation of Physiology to Physics and Chemistry." Dr. Haldane's address, we learn from the *Times*, will be an attempt to show that the physico-chemical theory of life, which seems still to be held by the majority of physiologists, is untenable as a scientific working hypothesis, and that biology must make fundamental assumptions which, at the present stage of our knowledge, differentiate it completely from physics and chemistry. He will endeavour to show that a meeting-point between biology and the physical sciences can only be looked for when biological conceptions are extended into the present sphere of physical and chemical conceptions. The relations of physiology to psychology and ethics will also be touched upon.

The Revised Regulations for the M.B., B.S., Degrees of the University of London recently adopted by the Senate, have now been published by the University.

Major Frederick McDowell, of the Royal Army Medical Corps, who died at Peshawar on August 6, served in the North-West Frontier of India in 1897-8, obtaining the medal with clasp for his services. During the South African war he was present at the operations in the Orange River Colony, including the actions at Biddulphsberg and Wittebergen, and also in the Transvaal from 1900 to 1902. For his services with the forces Major McDowell obtained the Queen's medal with three clasps and the King's medal with two clasps. He qualified L.R.C.P., L.R.C.S.Ed., and L.F.P.S. Glasg. in 1889.

According to the *Times* New York Correspondent an ordinance directing the pasteurising of all milk from cattle not proved free from tuberculosis has been adopted by the Chicago City Government. For some years Mr. Nathan Straus has been conducting a propaganda for the suppression of that disease by advocating this method of solving the pure milk problem, in connection with which he has recently given demonstrations in London, Dublin, and the Continental capitals. The Chicago City Council readily agreed to adopt Mr. Straus's remedy by 62 votes to one, upon the strong recommendation of Dr. Evans, the Commissioner of Health ,who cited Mr. Straus's demonstrations in this country and in Europe as proving the efficacy of the method he advocated, and who quoted the report of the British Royal Commission on Tuberculosis as well as high American authorities to show that tuberculosis can be communicated from cattle to human beings. A year ago the crusade for the pasteurisation of milk was defeated in New York owing, it is alleged, to the combined efforts of the Tammany political machine and the American Milk Trust.

### The Association of Public Vaccinators.

A special meeting of the Association of Public Vaccinators of England and Wales was held recently at Sheffield under the chairmanship of the President, Dr. Drury, of Halifax. The President drew attention to the fact that some boards of guardians treat the office of public vaccinator as a kind of bait or subsidy towards the under-paid medical officership. During the last few months several members of the Association have resigned their district medical officerships because of the ridiculous payment. In the Castleford area seven farthings per visit was the average paid to the medical officers for their services to the poor. When they resigned their posts they were immediately dismissed from the totally unconnected post of public vaccinator. Quite recently, in the Driffield district, the medical officer resigned his post because his rate of remuneration worked out at six farthings. He was also summarily dismissed from his post as public vaccinator, despite his twenty-four years' service. The President suggested that they should pass a resolution calling the attention of the Local Government Board to the matter, and that if the Board refused to deal with it the matter shall be brought before the notice of Parliament.

After considerable discussion a resolution was unanimously passed urging the Local Government Board to consider the hardships inflicted on public vaccinators. Subsequently an address was given by Dr. A. E. Cope laying down the outline of a suggested pamphlet for use at lectures or debates in defence of vaccination. The President, who advocated persuasion rather than compulsion in the matter of vaccination, suggested that upon the registration of the birth of a child the parent should be given an epitome of the conclusions of the majority report of the Royal Commission on Vaccination, which could be compressed into less space than Form A.

